# Two-Way Traffic in B Cell Development: Implications for Immune Tolerance

**DOI:** 10.1371/journal.pbio.0030112

**Published:** 2005-03-08

Development generally proceeds in one direction. Undifferentiated, pluripotent cells, which can become several different cell types, first of all become committed to restricted cell lineages. Then, under the control of developmental signals, committed cells gradually take on specialized characteristics, eventually producing mature, functioning cell types. To date, there has been little evidence to suggest that this process is ever reversed during normal development.

Now, however, Timothy Behrens and his colleagues report that the development of B lymphocytes, the antibody-producing cells of the immune system, can be switched into reverse by blocking or removing basal immunoglobulin signaling activity from immature B cells. Their findings have important implications for our understanding of how the immune system is tailored to respond efficiently to foreign antigens while ignoring self antigens and thus avoiding harmful autoimmune reactions.

B lymphocyte development, which occurs in the bone marrow, starts with the commitment of lymphoid progenitors to the B lineage and the somatic rearrangement of the heavy chain (HC) immunoglobulin (Ig) alleles. By stitching together diversity (D_H_), joining (J_H_), and variable (V_H_) region DNA segments, many pro-B cells, each with a single but unique HC allele, are produced. Those cells in which the stitched-together HC allele encodes a functional protein undergo clonal expansion and proceed to the pre-B stage, before repeating the whole rearrangement process for the light chain (LC) Ig alleles. A productive LC rearrangement results in surface expression of IgM, which acts as the B cell receptor (BCR) for antigen for the immature B cell.

During development, any B cells bearing strongly self-reactive Ig receptors are removed—this process is called tolerization—either by clonal deletion, by functional inactivation, or by receptor editing. In this last process, new LC rearrangements revise the antigen specificity of the receptor. Little is known about the mechanisms driving receptor editing, but these new data from Behrens and colleagues suggest that signals provided by surface BCRs might suppress receptor editing in immature B cells.

To test this hypothesis, the researchers used a genetic system to remove the BCR from the cell surface of immature B cells in an inducible manner in vitro, and then compared gene expression patterns in these cells, control immature B cells, and pre-B cells. They discovered that the BCR-deleted cells had a gene expression pattern similar to that of pre-B cells, indicating that the BCR-deleted cells had gone back to an earlier stage of B cell development as a consequence of losing their BCR.

The researchers saw a similar effect on B cell differentiation state when they blocked downstream signaling from the BCR by the use of the tyrosine kinase inhibitor herbimycin A or the phosphatidylinositol 3-kinase inhibitor wortmannin. Finally, the researchers showed that cells undergoing “back-differentiation” also restarted LC rearrangement or receptor editing.

These data, suggest Behrens and co-workers, indicate that immature B cells actively maintain their developmental state by constitutive basal Ig signaling through protein tyrosine kinases. Their findings, they say, throw new light onto how receptor editing might be regulated in immature B cells in order to ensure that tolerance to self antigens develops. The researchers propose that when immature B cells encounter self antigens, down-regulation of surface Ig (BCR) via endocytosis might induce a back-differentiation program and thus induce an efficient editing response to the self antigen. Further experiments are now needed to show that these processes do indeed happen not just in vitro, as revealed here, but also in vivo.

